# A Note on Arecoline

**Published:** 1899-09

**Authors:** J. C. Clemesha

**Affiliations:** M. D., M. R. C. S., Eng., L. R. C. P., London, Assistant surgeon Buffalo Eye and Ear Infirmary, ophthalmic surgeon to the Buffalo Hospital of the Sisters of Charity


### A NOTE ON ARECOLINE.

By J. C. CLEMESHA, M. D., M. R. C. S., Eng., L. R. C. P., London,
Assistant surgeon Buffalo Eye and Ear Infirmary, ophthalmic surgeon to the Buffalo Hospital of the Sisters of Charity.

ARECOLINE is one of the alkaloids found in the areca nut, the seeds of the areca catechu. Taken internally it causes vomiting and diarrhea and will be found useful in obstinate constipation from its action on the contractility of the intestine.

According to Frohner, arecoline is a sialagogue of the first rank, which is not only comparable to pilocarpine, but even exceeds it. Salivation occurs in about five minutes after its injection and attains its maximum in half an hour. Martin employed this drug as a teniafuge, using sixty grains of the powdered areca nut to obtain the desired effect and he observed the absence of colic.

The bromohydrate of arecoline is a white crystalline soluble salt, and when applied to the eye in the form of a one half or one per cent. aqueous solution, causes contraction of the pupil. A one half per cent. solution dropped into the conjunctival sac causes burning and slight conjunctival congestion. In from three to five minutes the pupil begins to contract and reaches its maximum in from ten to fifteen minutes, accompanied by spasm of the ciliary muscle. The maximum effect remains for a quarter of an hour or so, after which the pupil gradually returns to its normal condition. This is usually in the course of an hour or two.

The tension of the normal eye does not seem to be affected by it, but in cases of glaucoma, clinical results show this drug to be the equal of eserine.

Bietti observed that it appeared to act more promptly and more energetically than eserine, but that its effect was of shorter duration.

Finally, it keeps well in solution, retaining its active properties unchanged for an unlimited period.

329 Franklin Street.

---

To Remove the Odor of Iodoform.—Dr. Edwin Ricketts, of Cincinnati, says: "To do away with the 'smell' of iodoform that comes to the hands of the surgeon for handling it, I find that to rub on, after use of soap and water, a teaspoonful of vinegar (found in every household) does away, promptly, with the very disagreeable odor. —*Lancet-Clinic.*

